# Diets Rich in Fruits and Vegetables Are Associated with Lower Cardiovascular Disease Risk in Adolescents

**DOI:** 10.3390/nu10020136

**Published:** 2018-01-27

**Authors:** Kevan Mellendick, Lilly Shanahan, Laurie Wideman, Susan Calkins, Susan Keane, Cheryl Lovelady

**Affiliations:** 1 Department of Nutrition, University of North Carolina at Greensboro, Greensboro, NC, 27412, USA; calovela@uncg.edu; 2 Jacobs Center for Productive Youth Development, University of Zurich, CH-8050 Zurich, Switzerland; lilly.shanahan@uzh.ch; 3 Department of Kinesiology, University of North Carolina at Greensboro, Greensboro, NC, 27412, USA; l_widema@uncg.edu; 4 Department of Human Development & Family Studies, Department of Psychology, University of North Carolina at Greensboro, Greensboro, NC, 27412, USA; sdcalkin@uncg.edu; 5 Department of Psychology, University of North Carolina at Greensboro, Greensboro, NC, 27412, USA; spkeane@uncg.edu

**Keywords:** diet quality, healthy eating index, adolescent, cardiovascular disease, obesity

## Abstract

Obesity and cardiovascular disease (CVD) risk are public health concerns in adolescents, yet few studies have examined the association of their diet to CVD risk factors. This study investigated associations between diet, body mass index (BMI), waist circumference (WC), blood pressure (BP), and blood lipids in 163 16–17 year olds. Diet recall data were converted into Healthy Eating Index-2010 (HEI) to assess diet quality. Differences in diet between groups with normal or obese BMI, normal or hypertensive BP, and normal or altered lipids were determined. Associations between diet and BMI, WC, BP, and lipids, controlling for race, gender, and socioeconomic status, were examined. Mean HEI was 49.2 (±12.0), with no differences observed between groups. HEI was not associated with any CVD risk. Sweetened beverage consumption was higher in obese adolescents, and positively related to total cholesterol (TC). Fruit intake was negatively related to BMI and diastolic BP. Total vegetable intake was negatively related to systolic BP. Greens and beans were negatively related to TC and LDL. Whole grains were negatively related to HDL. This research suggests a cardioprotective effect of diets rich in fruits and vegetables, as well as low in sweetened beverages in adolescents.

## 1. Introduction

Although obesity rates in the United States appear to have plateaued among children and adolescents, diet quality continues to be poor. Depending on the source, 20.6% of U.S. adolescents, aged 12–19 years, are considered obese (>95th percentile for body mass index, BMI) and 33.6% are considered overweight (>85th percentile) [1,2]. Diet quality measurements consistently reveal well below optimal scores [3,4,5,6] in adolescents and longitudinal research indicates dietary patterns established in adolescence may persist into adulthood [7].

Abdominal obesity confers significant risk for metabolic dysfunction and cardiovascularrisk [8,9,10,11], regardless of body weight. Most studies use waist circumference (WC), as theindicator [12] of abdominal obesity, with the most common definition being greater than or equal to the 90th percentile of the sample [13]. In a recent review utilizing this cut point, a wide range of obesity rates have been found among 10–19-year-old subjects (range; 3.8% to 51.7%, depending on the country) [12].

Dyslipidemia and hypertension (HTN) are emerging health concerns among youth in the United States, especially among obese adolescents. In the US, approximately 20.3% of adolescents aged 12–19 have at least one abnormal lipid value, but the rate increases to 43.9% in obese adolescents [14]. Although incidence of HTN in this population is much lower at 2.5–4.5%, pre-HTN rates range from 10% to 15.7% [15,16,17,18,19]. These numbers suggest significant risk for the development of cardiovascular disease (CVD) in adolescents. However, with appropriate medical and/or lifestyle intervention, vascular changes are predominantly reversible at this stage of life [20,21]. With prior work identifying signs of CVD in teenagers [22], the adolescent age group seems to be an ideal target population for prevention efforts and identifying key CVD risks.

The influence of poor lifestyle habits, particularly diet, on the development of these CVD risk indicators has been studied thoroughly among adults, but not among adolescents. Of the adolescent research available, several single nutrients or clusters of nutrients have been implicated as contributors to adiposity, blood pressure (BP) or blood lipids [23,24,25,26]. Sugar, particularly in beverage form, has been implicated by multiple researchers for detrimental impact on adolescent weight, adiposity, and CVD risk [27,28,29,30,31,32,33,34,35]. Saturated fatty acid intake is linked to elevated BP and triglycerides (TGs) among adolescents [36,37], and total fat intake is related to elevated adiposity and low density lipoprotein (LDL) [37]. Dietary fiber intake, conversely, seems protective against CVD risks, as increasing fiber intake is related to decreasing visceral adiposity [38,39]. Certain food groups, particularly dairy, fruit, vegetable, nuts, seeds, and legumes, are also related to more favorable levels of body composition, total cholesterol (TC), LDL, and SBP [30,40,41,42,43,44]. Lastly, sodium intake has been linked with increasing systolic BP (SBP) among adolescents [45]. However, research on single nutrients dominates both the adult and adolescent literature. While some studies have examined food groups [23,30,40,41,42,43,44], there have been very few reports of the overall dietary intake of adolescents in relation to CVD risk.

Although single nutrient and food group research has yielded insights into the connection between diet and CVD risk, a validated overall diet quality measure, such as the HEI-2010 may be considered preferable, as it simultaneously encompasses multiple aspects of diet that may confound or offset the impact of specific nutrients or food groups. Minimal research exists examining diet quality with CVD risk in the adolescent population. The Healthy Eating Index-2010 (HEI-2010) based on the current Dietary Guidelines for Americans [46], is a tool for researchers to evaluate a diet, and may be more efficacious than single nutrient or dietary components to evaluate dietary impacts on health [3]. Data from NHANES show average HEI-2010 scores among US children and adolescents from 2003–2010 to be below 50 out of a possible 100, especially problematic when a score of 80 or higher is considered a “good” diet [3].

Most CVD risk research has focused on middle to older age adults and has identified decreased CVD mortality risk among adults with better diet quality [47]. The purpose of this study was to explore dietary intake and potential CVD risk factors in adolescence, a period in which early/initial stages of CVD may manifest. The overall aim of this study was to examine the relationships between diet quality and CVD risks in a group of 16–17 year old adolescents. The first objective was to determine if there were differences in diet quality (using the HEI-2010 and food groups) between adolescents with or without obesity, high BP, or dyslipidemia. The second objective was to determine if there were any significant associations between diet quality and CVD risk factors, controlling for race, gender and socioeconomic status.

## 2. Materials and Methods

The sample for this study was originally recruited at the age of two years from daycare centers, county health departments, and WIC programs in the greater Greensboro, NC community in 1997. The sample was also recruited with an effort to maintain approximately equivalent numbers of participants of each sex. Children with diagnosed developmental abnormalities or chronic diseases were excluded from the sample. It is a convenience sample, approximately 70% Caucasian, and the remainder predominantly African-American. Attrition from 1997 to time of data collection did not significantly alter the sample population in terms of gender, race, or socioeconomic status (SES).

In terms of SES, the sample is highly diverse. The Hollingshead social status score [48], a tool to estimate SES based on the parent’s education level and current employment, range among the sample from 9 to 66.

As part of this longitudinal research study, when participants reached adolescence, they were asked to return to the laboratory for health-related measurements (weight, height, waist circumference, biomarkers) and provide dietary recalls [49]. This study examined a subsample of 163 individuals who were 16–17 years old and completed the data collection procedures.

### 2.1. Human Subjects Protections

This study was approved by the Institutional Review Board of the University of North Carolina at Greensboro (IRB Approval 09-0427 (PI; Calkins) and 11-360 (PI; Wideman)). All parents/guardians of the minor participants provided consent and all adolescent participants provided assent.

### 2.2. Anthropometrics

A research assistant of the same gender as the participant measured the participant’s height, weight, and waist circumference. Height was measured with a Seca 222 stadiometer to the nearest 0.5 cm, with shoes off. Weight was measured via Seca 770 electronic scale to the nearest 0.01 kg. Waist circumference was measured with a Gulick tape measure directly against the skin, at the natural waist, the narrowest point of the abdomen between the lower rib cage and iliac crest.

Neither pubertal development nor Tanner scale was assessed for the participants.

### 2.3. Blood Pressure

Blood pressure was measured according to the National Heart, Lung, and Blood Institute standards [50]. The participant was seated upright in a comfortable chair, and blood pressure was measured using a sphygmomanometer and stethoscope. The participant rested 5 min in the chair prior to first measurement, and 5 min after the first measurement, prior to measuring a second time at the same site. The average of the measurements was used for analysis.

### 2.4. Blood Lipids

A trained research technician drew blood into SST Vacutainer^®^ type tubes (Becton, Dickinson, and Company, Franklin Lakes, NJ, USA). These tubes were gently inverted approximately ten times, then placed upright at room temperature for 20 min to allow for clotting. Tubes were centrifuged for 20 min at 3000 rpm at 4 °C. Serum was pipetted into microtubes in 500-microliter aliquots and stored at −80 °C until ready to be analyzed. Colorimetric assays were used to measure, total, HDL, and LDL cholesterol, and triglycerides using microtiter plate procedures outlined by the company (Wako Chemical, Richmond, VA, United States).

### 2.5. Diet Recalls

Prior to exiting the lab, a research assistant instructed the adolescent on the process of the diet recalls, and gave them a booklet of guides for portion sizes, which was to be utilized during the diet recall interview. Lastly, the research assistant ascertained the best contact telephone number and best times to contact the adolescent participant for the diet recalls.

Participants were called by trained and certified staff of the UNC-CH Nutrition Obesity Research Center to collect diet recalls on two weekdays and a weekend day using the multiple pass method. The multiple pass method includes several prompts, reviews, and follow on questions about food intake to improve recollection by the participant. The diet recalls collected were analyzed using the University of Minnesota’s Nutrition Data System for Research (NDSR) and standardized procedures outlined by the Nutrition Obesity Research Center’s program coordinator for quality assurance. Participants were given monetary incentives, with increasing amounts paid after each recall completed.

### 2.6. Diet Quality

Diet quality was assessed with the HEI-2010, a multi-component scoring tool to assess an individual’s diet quality. The HEI-2010 score measures diet quality based on the primary emphases of the 2010 Dietary Guidelines for Americans [46].

The HEI-2010 contains twelve-components, for a maximum score of 100 points. Each component can receive a score ranging from 0 for a minimum up to 20 points maximum (depending on assigned maximum point total). Scores between the minimum and maximum values are prorated based on intake. Scores above 80 are considered representative of a “good diet”, while scores of 50 or below are considered “poor”, and those between 50 and 80 are considered “needing improvement” [51].

Dietary data from NDSR was used to calculate the HEI-2010 score, based on average intake of foods and nutrients across all days’ dietary information obtained [52]. Generally, each average intake of a food group was converted to cup or ounce equivalents, as appropriate, then divided by average energy intake and multiplied by 1000 to standardize to an intake per 1000 kcals. An additional food group, sweetened beverages, was added to these data for additional analysis as other researchers have found it to be related to CVD risk factors. Sweetened beverages included beverages with added sugar such as soda, lemonade, Kool-Aid, sweet tea, sports drinks, and sweetened coffee drinks, but excluded fruit juice and flavored milk.

### 2.7. Socioeconomic Status

The Hollingshead Four Factor Index of Socioeconomic Status [48], a survey tool to estimate socio-economic status (SES) based on the parents’ education levels and current employment, was used to determine SES of the participants. The parental education level is scored on a scale of 1–7 (from <7th grade education to graduate degree earned), while parental occupation is rated on a scale of 1–9 (unskilled labor to major professional). The education score is multiplied by 3, the occupation score by 5, and the sum of the two multiples represents the total score, on a scale of 8–66.

### 2.8. Statistics

SPSS Statistics (Ver. 23; IBM, New York, United States) was used to perform all statistical analyses. Participant data was grouped based on categorical cutoffs identified for CVD risk, and classified as obese (BMI ≥ 95th percentile for age) vs. non-obese; elevated BP (BP ≥ 90th percentile for age) vs. normal BP; abnormal blood lipids (TC ≥ 200 mg/dL or LDL ≥ 130 mg/dL or TG ≥ 150 mg/dL or high density lipoprotein (HDL) ≤ 35 mg/dL) vs. normal blood lipids.

Descriptive statistics were computed for this sample. ANOVA was run comparing the means of HEI-2010 score and each HEI component score between obese and non-obese, hypertensive and normotensive participants, and dyslipidemic participants and those with normal blood lipids. Differences for all ANOVAs were considered significant at *p* < 0.05. Data was tested for normality, noting mild skew in all components, with no platykurtosis. As such, distributions were considered appropriate for ANOVA analyses, particularly considering prior research identifying minimal impact to false discovery rate with ANOVA in cases of moderate deviations from normality [53,54,55].

Stepwise linear regression analysis of HEI-2010 scores and food groups were run with all CVD risk variables: BMI, WC, SBP, diastolic BP (DBP), TC, TG, LDL, and HDL as outcome variables. Race, gender, and SES were covariates in the regression analyses. Correlations were considered significant at the *p* < 0.05 level.

## 3. Results

A total of 163 adolescents completed dietary recalls (three-day recalls (*n* = 149), two-day recalls (*n* = 6) and one-day recall (*n* = 8)). There were no significant differences in the average HEI scores nor any other dietary variables analyzed between the groups reporting one-, two or three-day averages. 

Anthropometric and blood pressure data were available for 143 individuals and an additional 19 self-reported height and weight data. Correlations between self-reported weight and height and weight and height measured in the lab were strong (*r* = 0.961, *p* < 0.001). Therefore, self-reported weight and height were used for those 19 participants who could not come to the lab for measurements. A total of 97 participants provided blood samples.

Table 1 highlights sample characteristics by BMI, HTN, and lipid status. Race was grouped as white and nonwhite (82% African American, 14% mixed race, 4% other race). Four individuals were considered underweight by BMI for age. A significantly higher proportion of nonwhite participants were obese by BMI cutoffs than white participants. Mean SES for the sample was 45.4 ± 13.1 (range of 9–66) with significant differences observed in SES between obese and non-obese participants. Racial differences in SES were also identified: non-white participants had significantly lower (40.5 ± 13.8) SES than white participants (47.6 ± 12.3, *p* < 0.01).

No significant differences were observed in rates of hypertension between genders, races, or by SES. Hypertensive participants had significantly higher BMI, WC, SBP, DBP, and TG compared to non-hypertensive participants. SBP and DBP were both significantly higher in the obese group compared to the non-obese group. The only significant difference observed in blood lipids by obesity status revealed a lower TC among those obese by BMI compared to non-obese. No significant differences were observed in rates of dyslipidemia by gender, race, or SES. Dyslipidemic participants had significantly higher LDL and TC than those with normal lipids, but no differences in HDL or TG. Additionally, no difference was observed in BMI between participants with dyslipidemia or not. Further analysis of classification for CVD risk, based on the TC: HDL ratio, identified 29.1% of adolescents in the above average risk for CVD (≥4.5 for females, ≥5.0 for males).

There were no gender differences in diet quality; however, there were racial differences. White participants had a higher HEI-2010 score compared to non-white participants (50.8 ± 12.6 vs. 46.0 ± 10.1, *p* < 0.01). Multiple component scores were different across race as well, with whole fruit (1.8 ± 1.9 vs. 0.9 ± 1.4), greens and beans (1.8 ± 2.1 vs. 1.2 ± 1.8), whole grains (4.0 ± 3.0 vs. 2.9 ± 2.9, at *p* < 0.01), dairy (6.9 ± 3.1 vs. 5.6 ± 2.8), and seafood and plant protein (1.9 ± 2.1 vs. 1.3 ± 1.8) scores all significantly higher in white participants compared to nonwhite participants.

Addressing our first objective, no significant differences in HEI-2010 total scores or component scores were observed between obese and non-obese groups (Table 2). However, sweetened beverage intake was significantly different, with more sweetened beverages consumed by the obese group, compared to the non-obese group (2.3 (±2.9) c/day vs. 1.4 (±1.5) c/day, respectively).

Continuing to examine our first objective, Table 3 reports the HEI scores among the total sample, hypertensive participants, and non-hypertensive participants. The only food component that was different between groups was the total protein group, with higher consumption in the hypertensive group compared to the non-hypertensive group. Moreover, no significant differences were observed in total HEI scores or rate of participants meeting recommendations for total HEI score among hypertensive and non-hypertensive participants.

Addressing the final aspect of our first objective, Table 4 shows differences observed in HEI scores among the total sample, dyslipidemic participants, and participants with normal blood lipids. No differences were observed in HEI component scores between dyslipidemic participants and those with normal blood lipids. However, among dyslipidemic participants, the rate of meeting the recommendation for greens and beans intake was significantly lower than those with normal blood lipids (13% vs. 33%).

An ANOVA comparing food group consumption across participants with elevated LDL found greater sweetened beverage consumption in the elevated LDL group (2.5 ± 3.0 c/day) than in the normal LDL group (1.6 ± 1.3 c/day). However, fewer sweetened beverages (1.0 ± 1.0 c/day) and added sugars (57.2 ± 33.2 g/day) were consumed by participants with elevated TG than by participants with normal TG (2.0 ± 2.1 c/day; 79.7 ± 50.5 g/day).

Examining our second objective, in stepwise linear regression analysis controlling for race, gender, and SES, diet quality (HEI) was not significantly related to any of the CVD risk factors. Table 5 shows the results of stepwise linear regression analysis of the components of HEI with BMI, WC, BP, and blood lipids. Examination of the components of HEI indicated that total fruit intake, was the only significant dietary predictor of BMI (B = −0.203); race (B = −0.270), but not SES (B = −0.047) or gender (B = −0.041), was also a significant predictor of BMI (R^2^ = 0.135). Total protein foods (B = −0.234) emerged as the only significant dietary predictor of WC, while race (B = −0.188) and gender (B = 0.303), but not SES (B = −0.101) were also significant (*R*^2^ = 0.125). Total vegetables (B = −0.198) was the only significant predictor of SBP, while race (B = −0.146), gender (B = 0.100), and SES (B = −0.054) were not significant (*R*^2^ = 0.077). Whole fruit (B = −0.193), total protein (B = −0.190), and gender (B = 0.245), but not race (B = −0.031) or SES (B = −0.158), were significant predictors of DBP (*R*^2^ = 0.126). Whole grains (B = −0.218) and SES (B = 0.229) were significant predictors of HDL, while race (B = 0.095) and gender (B = −0.047) were not significant predictors (*R*^2^ = 0.103). Greens and beans (B = −0.233) was the only significant predictor of LDL, with none of race (B = −0.132), gender (B = −0.147), or SES (B = 0.016) considered significant (*R*^2^ = 0.100). Greens and beans (B = −0.251), gender (B = −0.211), and SES (B = 0.238), but not race (B = −0.120), were significant predictors of TC (*R*^2^ = 0.144). The model for TG contained no significant predictors, and thus was excluded from Table 5.

## 4. Discussion

Among this diverse sample of 16–17-year-old adolescents, diet quality was similar regardless of obesity status (obese vs. non-obese), presence of hypertension (hypertensive vs. non-hypertensive), or presence of lipid abnormalities (dyslipidemic and normal blood lipids). Furthermore, diet quality was not predictive of any BMI, WC, BP, or blood lipids in this sample. However, it is important to note that the average diet quality score (as measured by HEI-2010) was 49.2, significantly below what would be considered a “good” diet score of 80 or higher (HEI ≥ 80).

Significant associations between individual dietary components and CVD risks were identified by stepwise linear regression while controlling for race, gender, and SES. Total vegetable intake was associated with lower SBP. Greater consumption of greens and beans was associated with lower TC and LDL levels. Meeting the recommended intake of greens and beans was more common in participants with normal blood lipids compared to those with dyslipidemia. Higher fruit consumption was related to lower BMI, while greater protein intake was associated with lower WC. Additionally, whole fruit and total protein food intake were both related to lower DBP. Protein intake was higher, however, in participants with hypertension compared to those without. More sweetened beverages were consumed by obese than by non-obese adolescents, and by adolescents with elevated LDL compared to those with normal LDL. However, fewer sweetened beverages and fewer added sugars were consumed by participants with elevated TG compared to normal TG. These significant components suggest a diet pattern higher in fruits and vegetables and lower in sweetened beverages is associated with a more favorable risk for obesity and CVD.

As noted above, the mean diet quality score of 49.2 was well below a score representative of a “good” diet, but it is very similar to values presented in other US research in this age group [3,4,5]. Previously reported mean HEI-2010 scores in this age group ranged from 43.59 in 14–18-year-old individuals to 49.8 in a group of 2–17 year olds [3,4,5]. The lack of differences in diet quality by BMI is consistent with previous work by Landy et al. [56] and Hurley et al. [57].

Moreover, this sample had similarly poor mean scores for multiple components of the HEI-2010 compared to previous research. In particular, the lowest mean scores for this sample were in the total fruit, whole fruit, greens and beans, whole grains, seafood and plant proteins, sodium, and refined grains components. Previous research indicates better average scores in total and whole fruit components, and lower scores in the total vegetable component than observed in this sample. Consistent with our findings, multiple studies demonstrate very low mean component scores in greens and beans, whole grains, seafood and plant proteins, sodium, and refined grains components among adolescents [4,58,59].

Findings from this research indicating greater intake of sweetened beverages among obese adolescents and negative relationship between fruit intake and BMI align with previous research [27,28,29,30,31,32,41]. While inverse relation between protein and WC is identified in some available research, the effect of protein consumption on WC is not well established [25,60].

Surprisingly, associations between adiposity and several other dietary components found in prior studies were not found in this work. In particular, several studies have reported inverse relations between BMI and/or WC with intake of vegetables and whole grains [61,62,63], which were not identified here. Notably, the most commonly consumed vegetable in this cohort was potato (in various forms), which may explain the lack of association between vegetables and BMI or WC. The impact of whole grain consumption on BMI or WC in this sample was likely limited by the low absolute quantity of whole grain consumed.

This study highlights differences in obesity status and diet quality across racial groups and SES. Particularly, non-white participants had markedly greater obesity rates than whites, and worse whole diet quality, specifically in terms of consuming less whole grain and dairy. In our sample, overweight and obese participants also had lower SES. These results align with previous studies, connecting lower SES [58,64,65,66] as well as African American (or nonwhite in the case of this sample) race [67,68,69,70,71] to poorer health outcomes, especially excess adiposity.

Rates of both hypertension and dyslipidemia in this sample were higher than those seen in other adolescent research. Compared to other US adolescent studies showing rates of HTN in the 2.5–4.5% [15,16,17,18,19], the rate in this sample was more than double at 10.6%. This may be a reflection of the region of the US in which participants were sampled. Previous work in adults has identified higher SBP and DBP, and greater incidence of HTN in the southeastern US, also known as the “stroke belt” [72,73]. Additionally, this sample had a much higher rate of participants with at least one abnormal blood lipid value at 72.2%, compared to the 20.3% rate found in a 2010 CDC report and the 42.9% rate among obese adolescents found in the same report [14]. More specifically, 51.5% of this sample had elevated TC. This strikingly high rate of elevated TC may be misleading in this sample, however, as mean HDL was over 60 mg/dL, creating a scenario in which many dyslipidemic participants do not have excessively high non-HDL cholesterol. In particular, at age 16, continuing growth, certain medication use (particularly birth control), and lack of exposure to substances such as cigarette smoke and androgens may favor higher HDL concentrations [74,75]. Upon further analysis, 70.1% of the sample had TC: HDL ratios predictive of below average heart disease risk (among adults), at <4.5 for females and <5.0 for males [76]. These factors suggest CVD risk related to blood lipids within this sample may be more similar to the national average, noted to be 20% in children aged 8–17 years [77], as measured by TC:HDL ratio. These discrepancies also reflect the substantive limitation of any single blood lipid marker in interpreting CVD risk in adolescents compared to the entirety of the blood lipid profile, especially in the absence of information about other confounding variables. In addition, prior research has noted that HDL subpopulations, which were not measured in this study, are better indicators of CVD risk than total HDL [78,79,80].

Many of the relationships between diet and CVD risk factors (including BP and lipids) identified in this study reflect those found in previous research in adolescents [42,44,81,82]. For instance, a variety of aspects of the DASH (Dietary Approaches to Stop Hypertension) diet were associated with lower SBP and DBP in this study: greater intake of total vegetables is associated with lower SBP, and greater intakes of whole fruit and greens and beans are related to DBP. Similar diet patterns were observed in this study in relation to blood lipids. A pattern of greater intake of greens and beans, whole fruit, and total vegetables, with lower sweetened beverage intake was related to lower TC and LDL.

However, several relations observed in this study conflict with results reported in prior literature. First, among participants with HTN, protein intake was higher in this study, which was not observed in previous research. Second, our work did not identify a link between BP and sodium [45], which was likely a function of consistently high sodium intake in this sample. Finally, higher sweetened beverage and added sugar consumption was related to lower TG, and higher whole grain intake was related to lower HDL. One possible explanation of these unexpected relations may be participation in athletics, as many athletes rely on and regularly consume sweetened sports drinks and refined carbohydrate foods as snacks before, during, or after practice. Athletes may have lower TG and higher HDL, even if consuming a diet high in refined grains and added sugars.

Despite some conflicts between the findings in this research and previous research, this research has several strengths. This study utilized gold standard methodology for collecting diet information, BP, and blood lipids, in a sample of socioeconomically diverse adolescents. Additionally, this study examined variables not commonly explored in this population, particularly BP, blood lipids, whole diet quality, and the associations between them. However, this research is limited in its observational design and relatively small sample size reflecting almost exclusively white and African American participants. As such, the results may not be generalizable to the whole US adolescent population. Additionally, this research is limited by a lack of full metabolic profile (such as blood glucose and c-reactive protein), body fat measurements, or physical activity data. This research adds to a limited body of evidence connecting diet to both dyslipidemia and HTN at a young age. Notably, the protective effects of many aspects of diet, particularly whole plant foods, are more consistently connected to BP and blood lipids than the negative impacts of other aspects of diet [42,44,81,82]. However, this research reflects the potential positive impact of greater intake of whole plant foods, particularly vegetables, fruits, and legumes on CVD risk reduction at a young age, as well as the negative impact of sweetened beverages and refined carbohydrates on CVD risk. This research also adds to a growing body of evidence indicating that young Americans are relying on diets high in calories, sodium, and sugar dense foods, with poor intakes of nutrient dense items, especially whole grains, fruits, vegetables, and legumes, thus resulting in poor overall diet quality. Considering dietary habits adopted in adolescence have been linked to dietary habits in adulthood [7], long term poor diet quality originating in youth could result in an increase in the prevalence of obesity as well as several chronic health conditions, such as hypertension, cardiovascular disease, and type 2 diabetes mellitus.

## Figures and Tables

**Table 1:** Sample characteristics by cardiovascular disease risk category.

		Obesity Status		Hypertension Status		Lipid Status	
	Total Sample	Non-Obese BMI	Obese BMI	Non-Hypertensive	Hypertensive	Normal Blood Lipids	Dyslipidemic
Gender							
Male, n, (%)	65 (40%)	53	11	50 (91%)	5 (9%)	14 (34%)	27 (66%)
Female, n, (%)	97 (60%)	83	14	77 (89%)	10 (11%)	13 (23%)	43 (77%)
Race							
White, n, (%)	106 (65%)	95 *	11 *	83 (89%)	10 (11%)	16 (27%)	44 (73%)
Nonwhite, n, (%)	56 (35%)	41 *	14 *	44 (90%)	5 (10%)	11 (30%)	26 (70%)
Socioeconomic Status (SES)	43.5 (14.4)	44.0 * (14.1)	41.3 * (16.6)	43.7 (14.3)	40.4 (16.0)	43.1 (15.5)	44.9 (12.5)
Body Mass Index (BMI), kg/m^2^	24.5 (6.3)	22.2 ^‡^ (3.1)	35.9 ^‡^ (6.2)	23.7 ^‡^ (5.4)	28.5 ^‡^ (8.7)	25.9 (7.5)	23.8 (5.2)
Waist Circumference (WC), cm	78.6 (14.2)	74.5 ^‡^ (8.4)	103.4 ^‡^ (17.1)	77.5 ^‡^ (12.7)	88.3 ^‡^ (22.0)	84.7 * (20.7)	77.0 * (11.3)
Systolic Blood Pressure (SBP), mmHg	114 (10)	113 ^‡^ (10)	120 ^‡^ (9)	112 ^‡^ (8)	130 ^‡^ (8)	115 (10)	113 (10)
Diastolic Blood Pressure (DBP), mmHg	69 (9)	68 ^‡^ (9)	76 ^‡^ (7)	68 ^‡^ (8)	79 ^‡^ (11)	69 (8)	69 (10)
High Density Lipoprotein (HDL), mg/dL	63 (22)	63 (23)	64 (17)	63 (22)	62 (24)	67 (11)	61 (25)
Low Density Lipoprotein (LDL), mg/dL	107 (39)	107 (40)	105 (32)	105 (38)	122 (45)	81 ^‡^ (18)	117 ^‡^ (40)
Total Cholesterol (TC), mg/dL	210 (45)	214 * (47)	187 * (22)	209 (44)	217 (57)	173 ^‡^ (19)	224 ^‡^ (44)
Triglycerides (TG), mg/dL	118 (49)	120 (51)	109 (37)	114 ^‡^ (46)	155 ^‡^ (61)	114 (34)	120 (54)

SES, BMI, WC, SBP, DBP, HDL, LDL, TC, and TG data expressed as mean (SD); SES measured by Hollingshead score, with a possible range of 8–66; * Indicates significant difference at *p* < 0.05; ^‡^ Indicates significant difference at *p* < 0.01.

**Table 2:** Healthy Eating Index-2010 scores of obese and non-obese adolescents by body mass index (BMI) for age.

	Score Range	Total Sample	Non-Obese BMI	Obese BMI	Recommendation/1000 kcal (Max Score)	Non-Obese Meeting Recommendation (%)	Obese Meeting Recommendation (%)
Total Fruit	0–5	1.5 (1.5)	1.5 (1.5)	1.1 (1.4)	≥0.8 c	5	4
Whole Fruit	0–5	1.5 (1.8)	1.5 (1.8)	1.4 (1.7)	≥0.4 c	9	8
Total Vegetables	0–5	2.7 (1.4)	2.7 (1.4)	2.7 (1.2)	≥1.1 c	12	4
Greens and Beans	0–5	1.6 (2.0)	1.7 (2.1)	1.3 (1.9)	≥0.4 c	18	16
Whole Grains	0–10	3.7 (3.0)	3.8 (3.0)	2.8 (2.8)	≥1.5 oz	7	4
Dairy	0–10	6.4 (3.1)	6.5 (3.1)	5.9 (3.1)	≥1.3 c	26	12
Total Protein Foods	0–5	4.4 (1.0)	4.3 (1.0)	4.7 (0.6)	≥2.5 oz	57	68
Seafood and Plant Proteins	0–5	1.7 (2.0)	1.7 (2.0)	1.6 (2.0)	≥0.8 oz	17	20
Fatty Acids	0–10	4.9 (3.1)	4.9 (3.2)	5.1 (2.6)	(MUFAs + PUFAs)/SFAs ≥ 2.5	10	4
Refined Grains	0-10	4.1 (3.4)	4.3 (3.5)	3.4 (3.0)	≤1.8 oz	9	0
Sodium	0–10	3.6 (2.9)	3.7 (3.0)	3.0 (2.8)	≤1.1 g	4	0
Empty Calories	0–20	13.1 (5.0)	13.3 (5.0)	12.3 (5.2)	≤19% of total kcal	12	12
Total HEI-2010 Score	0–100	49.2 (12.0)	50.0 (12.1)	45.2 (10.5)	≥80 (100)	1	0

Data expressed as mean (SD); No significant differences observed at *p* < 0.05 between Non-Obese and Obese by BMI.

**Table 3:** Healthy Eating Index-2010 (HEI-2010) scores of hypertensive (HTN) and normal blood pressure (Non-HTN) adolescents.

	Score Range	Total Sample	Non-HTN	HTN	Recommendation/1000 kcal (Max Score)	Non-HTN Meeting Recommendation (%)	HTN Meeting Recommendation (%)
Total Fruit	0–5	1.5 (1.5)	1.5 (1.5)	1.1 (1.1)	≥0.8 c	5	0
Whole Fruit	0–5	1.5 (1.8)	1.4 (1.7)	1.8 (2.0)	≥0.4 c	9	7
Total Vegetables	0–5	2.7 (1.3)	2.8 (1.4)	2.3 (1.0)	≥1.1 c	11	0
Greens and Beans	0–5	1.7 (2.0)	1.7 (2.0)	1.3 (1.9)	≥0.4 c	18	13
Whole Grains	0–10	3.6 (3.0)	3.5 (3.0)	4.4 (3.5)	≥1.5 oz	6	13
Dairy	0–10	6.5 (3.0)	6.4 (3.1)	7.5 (2.7)	≥1.3 c	22	40
Total Protein Foods	0–5	4.4 (1.0)	4.3 * (1.0)	4.9 * (0.2)	≥2.5 oz	57	67
Seafood and Plant Proteins	0–5	1.7 (2.0)	1.8 (2.0)	1.6 (2.0)	≥0.8 oz	17	20
Fatty Acids	0–10	4.9 (3.0)	4.8 (3.1)	5.1 (3.0)	(MUFAs + PUFAs)/SFAs ≥ 2.5	9	0
Refined Grains	0–10	4.1 (3.4)	4.1 (3.5)	4.3 (3.0)	≤1.8 oz	7	7
Sodium	0–10	3.5 (2.9)	3.6 (2.9)	2.9 (2.6)	≤1.1 g	2	0
Empty Calories	0–20	13.3 (4.7)	13.2 (4.8)	14.4 (3.8)	≤19% of total kcal	11	13
Total HEI-2010 Score	0–100	49.4 (11.8)	49.1 (11.8)	51.7 (11.8)	≥80 (100)	2	0

Data expressed as mean (SD); * Indicates significant difference at *p* < 0.05.

**Table 4:** Healthy Eating Index-2010 (HEI-2010) scores of dyslipidemic and adolescents with normal blood lipids.

	Score Range	Total Sample	Normal Blood Lipids	Dyslipidemic	Recommendation/1000 kcal (Max Score)	Normal Blood Lipids Meeting Recommendation (%)	Dyslipidemic Meeting Recommendation (%)
Total Fruit	0–5	1.5 (1.5)	1.7 (1.7)	1.3 (1.4)	≥0.8 c	7	1
Whole Fruit	0–5	1.5 (1.8)	1.6 (1.9)	1.3 (1.7)	≥0.4 c	15	4
Total Vegetables	0–5	2.7 (1.3)	2.8 (1.2)	2.7 (1.4)	≥1.1 c	7	13
Greens and Beans	0–5	1.7 (2.0)	2.3 (2.3)	1.4 (1.9)	≥0.4 c	33 *	13 *
Whole Grains	0–10	3.6 (3.0)	3.2 (2.5)	3.3 (3.0)	≥1.5 oz	4	6
Dairy	0–10	6.5 (3.0)	7.1 (2.6)	6.0 (3.2)	≥1.3 c	26	20
Total Protein Foods	0–5	4.4 (1.0)	4.4 (1.0)	4.4 (1.0)	≥2.5 oz	63	59
Seafood and Plant Proteins	0–5	1.7 (2.0)	1.7 (1.9)	1.5 (2.0)	≥0.8 oz	11	16
Fatty Acids	0–10	4.9 (3.0)	4.6 (2.9)	4.9 (3.1)	(MUFAs + PUFAs)/SFAs ≥ 2.5	7	9
Refined Grains	0–10	4.1 (3.4)	4.1 (3.0)	4.0 (3.4)	≤1.8 oz	7	7
Sodium	0–10	3.5 (2.9)	4.1 (3.2)	3.4 (2.7)	≤1.1 g	0	3
Empty Calories	0–20	13.3 (4.7)	13.1 (4.8)	13.3 (4.9)	≤19% of total kcal	4	11
Total HEI-2010 Score	0–100	49.4 (11.8)	50.6 (9.2)	47.4 (10.8)	≥80 (100)	0	0

Data expressed as mean (SD); * Indicates significant difference at *p* < 0.05.

**Table 5:** Relationships of Healthy Eating Index-2010 dietary components with body mass index, blood pressure, and blood lipids.

Dependent Variables	Independent Variables	Beta	*R*^2^
BMI	Race (0 nonwhite, 1 white)	−0.270 *	0.135
	Gender (0 female, 1 male)	−0.041	
	SES	−0.047	
	Total Fruit (c)	−0.203 *	
WC	Race (0 nonwhite, 1 white)	−0.188 *	0.125
	Gender (0 female, 1 male)	0.303 *	
	SES	−0.101	
	Total Protein (oz eq.)	−0.234 *	
SBP	Race (0 nonwhite, 1 white)	−0.146	0.077
	Gender (0 female, 1 male)	0.100	
	SES	−0.054	
	Total Vegetables (c)	−0.198 *	
DBP	Race (0 nonwhite, 1 white)	−0.031	0.126
	Gender (0 female, 1 male)	0.245 *	
	SES	−0.158	
	Whole Fruit (c)	−0.193 *	
	Total Protein (oz eq.)	−0.190 *	
HDL	Race (0 nonwhite, 1 white)	0.095	0.103
	Gender (0 female, 1 male)	−0.047	
	SES	0.229 *	
	Whole Grains (oz eq.)	−0.218 *	
LDL	Race (0 nonwhite, 1 white)	−0.132	0.100
	Gender (0 female, 1 male)	−0.147	
	SES	0.016	
	Greens and Beans (c)	−0.233 *	
TC	Race (0 nonwhite, 1 white)	−0.120	0.144
	Gender (0 female, 1 male)	−0.211 *	
	SES	0.238 *	
	Greens and Beans (c)	−0.251 *	

* Indicates significant difference at *p* < 0.05.

## References

[1] Ogden C.L., Carroll M.D., Kit B.K., Flegal K.M. (2012). Prevalence of obesity and trends in body mass index among us children and adolescents, 1999–2010. JAMA.

[2] Hales C.M., Carroll M.D., Fryar C.D., Ogden C.L. (2017). Prevalence of Obesity among Adults and Youth: United States, 2015–2016.

[3] Hiza H.A.B., Guenther P.M., Rihane C.I. (2013). Diet Quality of Children Age 2–17 Years as Measured by the Healthy Eating Index-2010.

[4] Fungwe T., Guenther P.M., Juan W., Hiza H.A.B., Lino M. (2009). The Quality of Children’s Diets in 2003–04 as Measured by the Healthy Eating Index-2005.

[5] O’Neil C.E., Nicklas T.A., Zanovec M., Fulgoni V.L. (2011). Diet quality is positively associated with 100% fruit juice consumption in children and adults in the United States: NHANES 2003–2006. Nutr. J..

[6] Beydoun M.A., Powell L.M., Chen X., Wang Y. (2011). Food prices are associated with dietary quality, fast food consumption, and body mass index among U.S. children and adolescents. J. Nutr..

[7] Mikkilä V., Räsänen L., Raitakari O.t., Pietinen P., Viikari J. (2005). Consistent dietary patterns identified from childhood to adulthood: The cardiovascular risk in Young Finns Study. Br. J. Nutr..

[8] Kaur J. (2014). A comprehensive review on metabolic syndrome. Cardiol. Res. Pract..

[9] Bastien M., Poirier P., Lemieux I., Després J.-P. (2014). Overview of epidemiology and contribution of obesity to cardiovascular disease. Prog. Cardiovasc. Dis..

[10] He F., Rodriguez-Colon S., Fernandez-Mendoza J., Vgontzas A.N., Bixler E.O., Berg A., Imamura Kawasawa Y., Sawyer M.D., Liao D. (2015). Abdominal obesity and metabolic syndrome burden in adolescents—Penn State Children Cohort study. J. Clin. Densitom..

[11] Després J.-P., Lemieux I., Bergeron J., Pibarot P., Mathieu P., Larose E., Rodés-Cabau J., Bertrand O.F., Poirier P. (2008). Abdominal obesity and the metabolic syndrome: Contribution to global cardiometabolic risk. Arterioscler. Thromb. Vasc. Biol..

[12] De Moraes A.C.F., Fadoni R.P., Ricardi L.M., Souza T.C., Rosaneli C.F., Nakashima A.T.A., Falcão M.C. (2011). Prevalence of abdominal obesity in adolescents: A systematic review. Obes. Rev..

[13] Cook S., Weitzman M., Auinger P., Nguyen M., Dietz W.H. (2003). Prevalence of a metabolic syndrome phenotype in adolescents: Findings from the third National Health and Nutrition Examination Survey, 1988–1994. Arch. Pediatr. Adolesc. Med..

[14] Centers for Disease Control and Prevention (CDC) (2010). Prevalence of abnormal lipid levels among youths-United States, 1999–2006. MMWR Morb. Mortal. Wkly. Rep..

[15] May A.L., Kuklina E.V., Yoon P.W. (2012). Prevalence of cardiovascular disease risk factors among US adolescents, 1999−2008. Pediatrics.

[16] Ostchega Y., Carroll M., Prineas R.J., McDowell M.A., Louis T., Tilert T. (2009). Trends of elevated blood pressure among children and adolescents: Data from the National Health and Nutrition Examination Survey 1988–2006. Am. J. Hypertens..

[17] McNiece K.L., Poffenbarger T.S., Turner J.L., Franco K.D., Sorof J.M., Portman R.J. (2007). Prevalence of hypertension and pre-hypertension among adolescents. J. Pediatr..

[18] Acosta A.A., Samuels J.A., Portman R.J., Redwine K.M. (2012). Prevalence of persistent prehypertension in adolescents. J. Pediatr..

[19] Sorof J.M., Lai D., Turner J., Poffenbarger T., Portman R.J. (2004). Overweight, ethnicity, and the prevalence of hypertension in school-aged children. Pediatrics.

[20] Olsen R. (2000). Atherogenesis in children: Implications for the prevention of athersclerosis. Adv. Pediatr..

[21] Celermajer D.S. (1997). Endothelial dysfunction: Does it matter? Is it reversible?. J. Am. Coll. Cardiol..

[22] Berenson G.S., Srinivasan S.R., Bao W., Newman W.P., Tracy R.E., Wattigney W.A. (1998). Association between multiple cardiovascular risk factors and atherosclerosis in children and young adults. The Bogalusa Heart study. N. Engl. J. Med..

[23] Epstein L.H., Gordy C.C., Raynor H.A., Beddome M., Kilanowski C.K., Paluch R. (2001). Increasing fruit and vegetable intake and decreasing fat and sugar intake in families at risk for childhood obesity. Obesity.

[24] Ludwig D.S., Peterson K.E., Gortmaker S.L. (2001). Relation between consumption of sugar-sweetened drinks and childhood obesity: A prospective, observational analysis. Lancet.

[25] Ludwig D.S., Pereira M.A., Kroenke C.H. (1999). Dietary fiber, weight gain, and cardiovascular disease risk factors in young adults. JAMA.

[26] Pereira M.A., Kartashov A.I., Ebbeling C.B., Van Horn L., Slattery M.L., Jacobs D.R. Jr., Ludwig D.S. (2005). Fast-food habits, weight gain, and insulin resistance (the CARDIA study): 15-year prospective analysis. Lancet.

[27] Collison K.S., Zaidi M.Z., Subhani S.N., Al-Rubeaan K., Shoukri M., Al-Mohanna F.A. (2010). Sugar-sweetened carbonated beverage consumption correlates with BMI, waist circumference, and poor dietary choices in school children. BMC Public Health.

[28] Striegel-Moore R.H., Thompson D., Affenito S.G., Franko D.L., Obarzanek E., Barton B.A., Schreiber G.B., Daniels S.R., Schmidt M., Crawford P.B. (2006). Correlates of beverage intake in adolescent girls: The National Heart, Lung, and Blood Institute Growth and Health Study. J. Pediatr..

[29] Libuda L., Alexy U., Sichert-Hellert W., Stehle P., Karaolis-Danckert N., Buyken A.E., Kersting M. (2008). Pattern of beverage consumption and long-term association with body-weight status in German adolescents—Results from the DONALD study. Br. J. Nutr..

[30] Francis D.K., Van den Broeck J., Younger N., McFarlane S., Rudder K., Gordon-Strachan G., Grant A., Johnson A., Tulloch-Reid M., Wilks R. (2009). Fast-food and sweetened beverage consumption: Association with overweight and high waist circumference in adolescents. Public Health Nutr..

[31] Fiorito L.M., Marini M., Francis L.A., Smiciklas-Wright H., Birch L.L. (2009). Beverage intake of girls at age 5 y predicts adiposity and weight status in childhood and adolescence. Am. J. Clin. Nutr..

[32] Gillis L.J., Bar-Or O. (2003). Food away from home, sugar-sweetened drink consumption and juvenile obesity. J. Am. Coll. Nutr..

[33] Nguyen S., Choi H.K., Lustig R.H., Hsu C. (2009). Sugar-sweetened beverages, serum uric acid, and blood pressure in adolescents. J. Pediatr..

[34] Martin-Calvo N., Martínez-González M.-A., Bes-Rastrollo M., Gea A., Ochoa M.C., Marti A., GENOI Members (2014). Sugar-sweetened carbonated beverage consumption and childhood/adolescent obesity: A case-control study. Public Health Nutr..

[35] Pollock N.K., Bundy V., Kanto W., Davis C.L., Bernard P.J., Zhu H., Gutin B., Dong Y. (2012). Greater fructose consumption is associated with cardiometabolic risk markers and visceral adiposity in adolescents. J. Nutr..

[36] Niinikoski H., Jula A., Viikari J., Rönnemaa T., Heino P., Lagström H., Jokinen E., Simell O. (2009). Blood pressure is lower in children and adolescents with a low-saturated-fat diet since infancy. The Special Turku Coronary Risk Factor Intervention Project. Hypertension.

[37] Washi S.A., Ageib M.B. (2010). Poor diet quality and food habits are related to impaired nutritional status in 13- to 18-year-old adolescents in Jeddah. Nutr. Res..

[38] Carlson J.J., Eisenmann J.C., Norman G.J., Ortiz K.A., Young P.C. (2011). Dietary fiber and nutrient density are inversely associated with the metabolic syndrome in US adolescents. J. Am. Diet. Assoc..

[39] Davis J.N., Alexander K.E., Ventura E.E., Toledo-Corral C.M., Goran M.I. (2009). Inverse relation between dietary fiber intake and visceral adiposity in overweight Latino youth. Am. J. Clin. Nutr..

[40] Kelishadi R., Ardalan G., Gheiratmand R., Gouya M.M., Razaghi E.M., Delavari A., Majdzadeh R., Heshmat R., Motaghian M., Barekati H. (2007). Association of physical activity and dietary behaviours in relation to the body mass index in a national sample of Iranian children and adolescents: CASPIAN study. Bull. World Health Organ..

[41] Bradlee M.L., Singer M.R., Qureshi M.M., Moore L.L. (2010). Food group intake and central obesity among children and adolescents in the Third National Health and Nutrition Examination Survey (NHANES III). Public Health Nutr..

[42] Bradlee M.L., Singer M.R., Daniels S.R., Moore L.L. (2013). Eating patterns and lipid levels in older adolescent girls. Nutr. Metab. Cardiovasc. Dis..

[43] Moore L.L., Singer M.R., Qureshi M.M., Bradlee M.L. (2008). Dairy intake and anthropometric measures of body fat among children and adolescents in NHANES. J. Am. Coll. Nutr..

[44] Bel-Serrat S., Mouratidou T., Jiménez-Pavón D., Huybrechts I., Cuenca-García M., Mistura L., Gottrand F., González-Gross M., Dallongeville J., Kafatos A. (2014). Is dairy consumption associated with low cardiovascular disease risk in European adolescents? Results from the HELENA study. Pediatr. Obes..

[45] He F.J., Marrero N.M., MacGregor G.A. (2007). Salt and blood pressure in children and adolescents. J. Hum. Hypertens..

[46] Guenther P.M., Kirkpatrick S.I., Reedy J., Krebs-Smith S.M., Buckman D.W., Dodd K.W., Casavale K.O., Carroll R.J. (2014). The Healthy Eating Index-2010 is a valid and reliable measure of diet quality according to the 2010 Dietary Guidelines for Americans. J. Nutr..

[47] Onvani S., Haghighatdoost F., Surkan P.J., Larijani B., Azadbakht L. (2017). Adherence to the Healthy Eating Index and Alternative Healthy Eating Index dietary patterns and mortality from all causes, cardiovascular disease and cancer: A meta-analysis of observational studies. J. Hum. Nutr. Diet..

[48] Hollingshead A. (1975). Four Factor Index of Social Status.

[49] Wideman L., Calkins S.D., Janssen J.A., Lovelady C.A., Dollar J.M., Keane S.P., Perrin E.M., Shanahan L. (2016). Rationale, design and methods for the RIGHT Track Health study: Pathways from childhood self-regulation to cardiovascular risk in adolescence. BMC Public Health.

[50] National High Blood Pressure Education Program Working Group on High Blood Pressure in Children and Adolescents (2004). The fourth report on the diagnosis, evaluation, and treatment of high blood pressure in children and adolescents. Pediatrics.

[51] Guenther P.M., Casavale K.O., Reedy J., Kirkpatrick S.I., Hiza H.A.B., Kuczynski K.J., Kahle L.L., Krebs-Smith S.M. (2013). Update of the Healthy Eating Index: HEI-2010. J. Acad. Nutr. Diet..

[52] Miller P.E., Mitchell D.C., Harala P.L., Pettit J.M., Smiciklas-Wright H., Hartman T.J. (2011). Development and evaluation of a method for calculating the Healthy Eating Index-2005 using the Nutrition Data System for research. Public Health Nutr..

[53] Glass G.V., Peckham P.D., Sanders J.R. (1972). Consequences of failure to meet assumptions underlying the fixed effects analyses of variance and covariance. Rev. Educ. Res..

[54] Harwell M.R., Rubinstein E.N., Hayes W.S., Olds C.C. (1992). Summarizing monte carlo results in methodological research: The one- and two-factor fixed effects ANOVA cases. J. Educ. Stat..

[55] Lix L.M., Keselman J.C., Keselman H.J. (1996). Consequences of Assumption Violations Revisited: A quantitative review of alternatives to the one-way analysis of variance *F* Test. Rev. Educ. Res..

[56] Landy D.C., Lipsitz S.R., Kurtz J.M., Hinkle A.S., Constine L.S., Adams M.J., Lipshultz S.E., Miller T.L. (2013). Dietary quality, caloric intake, and adiposity of childhood cancer survivors and their siblings: An analysis from the cardiac risk factors in childhood cancer survivors study. Nutr. Cancer.

[57] Hurley K.M., Oberlander S.E., Merry B.C., Wrobleski M.M., Klassen A.C., Black M.M. (2009). The healthy eating index and youth healthy eating index are unique, nonredundant measures of diet quality among low-income, African American adolescents. J. Nutr..

[58] Hiza H.A.B., Casavale K.O., Guenther P.M., Davis C.A. (2013). Diet quality of Americans differs by age, sex, race/ethnicity, income, and education level. J. Acad. Nutr. Diet..

[59] Banfield E.C., Liu Y., Davis J.S., Chang S., Frazier-Wood A.C. (2016). Poor adherence to US dietary guidelines for children and adolescents in the national health and nutrition examination survey population. J. Acad. Nutr. Diet..

[60] Ambrosini G.L., Huang R.-C., Mori T.A., Hands B.P., O’Sullivan T.A., de Klerk N.H., Beilin L.J., Oddy W.H. (2010). Dietary patterns and markers for the metabolic syndrome in Australian adolescents. Nutr. Metab. Cardiovasc. Dis..

[61] Field A.E., Gillman M.W., Rosner B., Rockett H.R., Colditz G.A. (2003). Association between fruit and vegetable intake and change in body mass index among a large sample of children and adolescents in the United States. Int. J. Obes..

[62] Rolls B.J., Ello-Martin J.A., Tohill B.C. (2004). What can intervention studies tell us about the relationship between fruit and vegetable consumption and weight management?. Nutr. Rev..

[63] Tohill B.C., Seymour J., Serdula M., Kettel-Khan L., Rolls B.J. (2004). What epidemiologic studies tell us about the relationship between fruit and vegetable consumption and body weight. Nutr. Rev..

[64] Darmon N., Drewnowski A. (2008). Does social class predict diet quality?. Am. J. Clin. Nutr..

[65] Galobardes B., Morabia A., Bernstein M.S. (2001). Diet and socioeconomic position: Does the use of different indicators matter?. Int. J. Epidemiol..

[66] Lallukka T., Laaksonen M., Rahkonen O., Roos E., Lahelma E. (2006). Multiple socio-economic circumstances and healthy food habits. Eur. J. Clin. Nutr..

[67] Thompson D.R., Obarzanek E., Franko D.L., Barton B.A., Morrison J., Biro F.M., Daniels S.R., Striegel-Moore R.H. (2007). Childhood overweight and cardiovascular disease risk factors: The National Heart, Lung, and Blood Institute Growth and Health Study. J. Pediatr..

[68] Harshfield G.A., Alpert B.S., Willey E.S., Somes G.W., Murphy J.K., Dupaul L.M. (1989). Race and gender influence ambulatory blood pressure patterns of adolescents. Hypertension.

[69] Luft F.C., Miller J.Z., Grim C.E., Fineberg N.S., Christian J.C., Daugherty S.A., Weinberger M.H. (1991). Salt sensitivity and resistance of blood pressure. Age and race as factors in physiological responses. Hypertension.

[70] Daniels S.R., Khoury P.R., Morrison J.A. (1997). The utility of body mass index as a measure of body fatness in children and adolescents: differences by race and gender. Pediatrics.

[71] Wang C.Y., Gortmaker S.L., Taveras E.M. (2011). Trends and racial/ethnic disparities in severe obesity among US children and adolescents, 1976–2006. Int. J. Pediatr. Obes..

[72] Hajjar I., Kotchen T. (2003). Regional variations of blood pressure in the united states are associated with regional variations in dietary intakes: The NHANES-III data. J. Nutr..

[73] Obisesan T.O., Vargas C.M., Gillum R.F. (2000). Geographic variation in stroke risk in the United States. Region, urbanization, and hypertension in the Third National Health and Nutrition Examination survey. Stroke.

[74] Glazer G. (1991). Atherogenic effects of anabolic steroids on serum lipid levels: A literature review. Arch. Intern. Med..

[75] Connelly P., Petrasovits A., Stachenko S., MacLean D., Little J., Chockalingam A. (1999). Prevalence of high plasma triglyceride combined with low HDL-c levels and its association with smoking, hypertension, obesity, diabetes, sedentariness and LDL-c levels in the Canadian population. Canadian Heart Health Surveys Research Group. Can. J. Cardiol..

[76] Millán J., Pintó X., Muñoz A., Zúñiga M., Rubiés-Prat J., Pallardo L.F., Masana L., Mangas A., Hernández-Mijares A., González-Santos P. (2009). Lipoprotein ratios: Physiological significance and clinical usefulness in cardiovascular prevention. Vasc. Health Risk Manag..

[77] Kit B.K., Kuklina E., Carroll M.D., Ostchega Y., Freedman D.S., Ogden C.L. (2015). Prevalence of and trends in dyslipidemia and blood pressure among US children and adolescents, 1999–2012. JAMA Pediatr..

[78] Mascarenhas-Melo F., Sereno J., Teixeira-Lemos E., Marado D., Palavra F., Pinto R., Rocha-Pereira P., Teixeira F., Reis F. (2013). Implication of low HDL-c levels in patients with average LDL-c levels: A focus on oxidized LDL, large HDL subpopulation, and adiponectin. Mediat. Inflamm..

[79] Mascarenhas-Melo F., Sereno J., Teixeira-Lemos E., Ribeiro S., Rocha-Pereira P., Cotterill E., Teixeira F., Reis F. (2013). Markers of increased cardiovascular risk in postmenopausal women: Focus on oxidized-LDL and HDL subpopulations. Dis. Markers.

[80] Mascarenhas-Melo F., Marado D., Palavra F., Sereno J., Coelho Á., Pinto R., Teixeira-Lemos E., Teixeira F., Reis F. (2013). Diabetes abrogates sex differences and aggravates cardiometabolic risk in postmenopausal women. Cardiovasc. Diabetol..

[81] Mohseni-Takalloo S., Mirmiran P., Hosseini-Esfahani F., Mehrabi Y., Azizi F. (2014). Metabolic syndrome and its association with Healthy Eating Index-2005 in adolescents: Tehran lipid and glucose study. J. Food Nutr. Res..

[82] Moore L.L., Singer M.R., Bradlee M.L., Djouss L., Proctor M.H., Cupples L.A., Ellison R.C. (2005). Intake of fruits, vegetables, and dairy products in early childhood and subsequent blood pressure change. Epidemiology.

